# Formononetin Upregulates Nrf2/HO-1 Signaling and Prevents Oxidative Stress, Inflammation, and Kidney Injury in Methotrexate-Induced Rats

**DOI:** 10.3390/antiox8100430

**Published:** 2019-09-26

**Authors:** Saleem H. Aladaileh, Omnia E. Hussein, Mohammad H. Abukhalil, Sultan A. M. Saghir, May Bin-Jumah, Manal A. Alfwuaires, Mousa O. Germoush, Amer A. Almaiman, Ayman M. Mahmoud

**Affiliations:** 1 Department of Medical Analysis, Princess Aisha Bint Al-Hussein Faculty of Nursing and Health Sciences, Al-Hussein Bin Talal University, Ma`an 71111, Jordan; sadaileh@ahu.edu.jo (S.H.A.); sultan.s.ayesh@ahu.edu.jo (S.A.M.S.); 2 Department of Biology, Faculty of Science, Al-Hussein Bin Talal University, Ma`an 71111, Jordan; 3 Physiology Division, Department of Zoology, Faculty of Science, Beni-Suef University, Beni-Suef 62514, Egypt; omniaaa411@yahoo.com; 4 Department of Biology, College of Science, Princess Nourah bint Abdulrahman University, Riyadh 84428, Saudi Arabia; mnbinjumah@pnu.edu.sa; 5 Department of Biology, Faculty of Science, King Faisal University, Al-Ahsa 31982, Saudi Arabia; malfwuaires@kfu.edu.sa; 6 Department of Biology, College of Science, Jouf University, Sakaka 2014, Saudi Arabia; germoush@ju.edu.sa; 7 Department of Applied Medical Sciences, Community College of Unaizah, Qassim University, Buraydah 51431, Saudi Arabia; ameralmeman@hotmail.com

**Keywords:** formononetin, methotrexate, ROS, Nrf2, nephrotoxicity

## Abstract

Acute kidney injury (AKI) is a serious complication of methotrexate (MTX). This study explored the protective effect of the isoflavone formononetin (FN) against MTX nephrotoxicity with an emphasis on oxidative stress, inflammation, and nuclear factor (erythroid-derived 2)-like 2/heme oxygenase 1 (Nrf2/HO-1) signaling. Rats received FN (10, 20, and 40 mg/kg) for 10 days and a single dose of MTX on day 7. MTX induced kidney injury was characterized by increased serum creatinine and urea, kidney injury molecule-1 (Kim-1), and several histological alterations. FN ameliorated kidney function and inhibited the renal tissue injury induced by MTX. Reactive oxygen species (ROS), lipid peroxidation (LPO), nitric oxide, and 8-Oxo-2′-deoxyguanosine were increased, whereas antioxidant defenses were diminished in the kidney of MTX-administered rats. In addition, MTX upregulated renal iNOS, COX-2, TNF-α, IL-1β, Bax, caspase-9, and caspase-3, and decreased Bcl-2, Nrf2, and HO-1. FN suppressed oxidative stress, LPO, DNA damage, iNOS, COX-2, proinflammatory cytokines, and apoptosis, and boosted Bcl-2, antioxidants, and Nrf2/HO-1 signaling in MTX-administered rats. In conclusion, FN prevents MTX-induced AKI by activating Nrf2/HO-1 signaling and attenuates oxidative damage and inflammation. Thus, FN may represent an effective adjuvant that can prevent MTX nephrotoxicity, pending further mechanistic studies.

## 1. Introduction

Methotrexate (MTX), a folic acid antagonist, is a potent chemotherapeutic agent used in the treatment of malignancies and inflammatory diseases [1]. However, some restrictions have been made on its clinical applications because of its nephrotoxicity and other adverse effects [2,3,4]. Renal dysfunction is thought to be provoked by the precipitation of MTX, 7-hydroxy MTX, and 2, 4-diamino-N10-methylpteroic acid in the renal tubules, resulting in cell death and consequent infiltration of inflammatory cells [3,5]. Although the mechanism of MTX nephrotoxicity is not fully understood, it has been reported to involve excess production of reactive oxygen species (ROS), inflammation, mitochondrial dysfunction, DNA damage, and caspase activation, eventually culminating in renal dysfunction [4,6,7,8].

One of the untoward effects of MTX is its ability to promote ROS production, leading to inflammation and cell death. As reported previously, MTX has the ability to increase the production of ROS through provoking nicotinamide adenine dinucleotide phosphate (NADPH) depletion [9], neutrophils activation [10], and suppression of homocysteine remethylation [11]. Excess ROS can activate nuclear factor-kappaB (NF-κB) and the release of proinflammatory cytokines [12], and trigger mitochondrial dysfunction and apoptosis [13]. Given the role of ROS and oxidative stress in the development of MTX nephrotoxicity, the induction of antioxidant and cytoprotective enzymes is critical. Indeed, the cell is well equipped with a variety of antioxidants that serve to protect against various redox insults. Multiple lines of evidence suggested a key role for nuclear factor (erythroid-derived 2)-like 2 (Nrf2) in the cellular defense against oxidants and the regulation of expression of several antioxidant and cytoprotective genes [14]. Under physiological conditions, Kelch-like ECH associated protein 1 (Keap1) sequesters Nrf2 in the cytosol via binding to the N-terminal Neh2 domain. This binding facilitates Nrf2 degradation via the ubiquitin-dependent proteasomal system [15]. However, various oxidant and toxic insults disturb this sequestration, thereby inducing the nuclear translocation of Nrf2 and its binding to the antioxidant response element (ARE) and expression of antioxidant genes, including NADPH-quinone oxireductase 1 (NQO1) and heme oxygenase 1 (HO-1) [15,16]. Nrf2 signaling activation has been implicated in ameliorating drug-induced oxidative stress and organ damages [6,17,18,19,20]. On the contrary, Nrf2 deficiency was associated with a greater severity of renal injury in a mouse model of ischemic and nephrotoxic acute kidney injury (AKI) [21].

Several studies have demonstrated the renoprotective effects of various antioxidants in animal models of AKI [6,7,22]. Formononetin (FN, 7-Hydroxy-4′-methoxyisoflavone) is a bioactive isoflavone constituent of the red clover (*Trifolium pratense*) and *Astragalus membranaceus* [23]. It has been shown to have numerous medicinal benefits, such as antioxidant, anti-inflammatory, and antitumor activities [23,24,25,26]. FN prevented cisplatin-induced AKI and inhibited apoptosis of renal tubular cells [27], and attenuated lipopolysaccharide (LPS)-induced inflammation and lung injury in mice [25]. In addition, FN was found to protect pancreatic β-cell against apoptosis by inhibiting the activation of NF-κB and reducing nitric oxide (NO) generation in rat insulinoma cell line [28]. A recent study showed that FN maintained kidney function by inhibiting ROS overproduction and restoration of antioxidants in a rat model of diabetic nephropathy [29]. Furthermore, FN exhibited antitumoral actions in a myeloma model through the regulation of multiple oncogenic cascades and gene products [23]. Despite the described biological activities of FN, its ability to ameliorate MTX-induced AKI has not been demonstrated yet. We investigated the protective effect of FN against AKI in MTX-administered rats, pointing to its ability to suppress oxidative stress and inflammation, and the role of Nrf2 signaling.

## 2. Materials and Methods

### 2.1. Experimental Animals and Treatments

Thirty-six male Wistar rats weighing 160 to 180g were used to investigate the nephroprotective effect of FN. The animals, obtained from VACSERA (Giza, Egypt), were housed under standard conditions and allowed free access to food and water. Protocols involving the use of animals were performed in line with the guidelines of the National Institutes of Health (NIH publication No. 85-23, revised 2011), and approved by the local ethical committee (2019/198).

According to the study protocol, six groups of rats (*n* = 6 rats in each group) were employed to study the renoprotective potential of FN as follows:

Group I (Control): Rats received 0.5% carboxymethyl cellulose (CMC) orally for 10 consecutive days.

Group II (FN): Rats received FN (40 mg/kg) [29] orally for 10 days.

Group III (MTX): Rats received 0.5% CMC orally for 10 days and MTX at day 7.

Group IV (MTX + 10 mg FN): Rats received 10 mg/kg FN [29] orally for 10 days and MTX at day 7.

Group V (MTX + 20 mg FN): Rats received 20 mg/kg FN [29] orally for 10 days and MTX at day 7.

Group VI (MTX + 40 mg FN): Rats received 40 mg/kg FN [29] orally for 10 days and MTX at day 7.

MTX (Shanxi PUDE, China) was dissolved in saline and injected intraperitoneally (i.p.) at 20 mg/kg dose [6]. Groups I and II received a single i.p. injection of saline on day 7. FN (Sigma, St. Louis, MO, USA) was dissolved in 0.5% CMC. At the 11^th^ day, the animals were sacrificed, and blood and kidney samples were harvested for further investigations. Serum was prepared from the blood samples, and the kidneys were washed in cold phosphate buffered saline (PBS). Specimens from the kidneys were fixed in 10% neutral buffered formalin while others were kept frozen at −80°C. Other samples were homogenized (10% *w/v*) in PBS supplemented with 0.5 mM EDTA and proteinase inhibitors, centrifuged, and the clear supernatant was collected.

### 2.2. Biochemical Assays

#### 2.2.1. Determination of Creatinine and Urea

Serum creatinine [30] and urea [31] were measured using reagent kits supplied by Spinreact (Girona, Spain).

#### 2.2.2. Determination of Kim-1, Caspases and Cytokines

Kidney injury molecule-1 (Kim-1), caspase-3, and caspase-9 were assayed using ELISA kits purchased from Cusabio (Wuhan, China). Tumor necrosis factor alpha (TNF-α) and interleukin (IL)-1β were assayed using R&D Systems ELISA kits (Minneapolis, MN, USA).

#### 2.2.3. Determination of Oxidative Stress Markers and Antioxidants

ROS were measured immediately in the tissue homogenates using 2′,7′-dichlorodihydrofluorescein diacetate (H2DCF-DA), as previously described [32]. Thiobarbituric acid reactive species (TBARS) were measured as a lipid peroxidation (LPO) marker [33], and NO was determined using Griess reagent [34]. 8-Oxo-2′-deoxyguanosine (8-Oxo-dG) was assayed by Cusabio kit (Wuhan, China) following the supplied instructions. Reduced glutathione (GSH) [35], and the antioxidant enzymes superoxide dismutase (SOD) [36], catalase (CAT) [37], and glutathione peroxidase (GPx) [38] were assayed in the renal tissue homogenates.

#### 2.2.4. Determination of HO-1 Activity

Total HO-1 activity was measured following the method of Abraham et al. [39]. Briefly, tissue samples were mixed with 2 mM glucose-6-phosphate, 0.8 mM NADPH, 20 µM hemin, and 0.2 U glucose-6-phosphate dehydrogenase in a total volume of 1.2 mL. After incubation at 37 °C for 1 h, the absorbance was measured at 464 nm, and the activity was normalized to the control group.

#### 2.2.5. Determination of ATP

Adenosine triphosphate (ATP) content in the renal homogenate was measured using a kit supplied by Sigma (St. Louis, MO, USA). In this test, ATP is determined by phosphorylating glycerol, and the product is proportional to the amount of ATP in the sample and is determined calorimetrically at 570 nm.

### 2.3. Histological Examination of Kidney Sections

Specimens from kidney fixed in 10% buffered formalin were dehydrated, embedded in paraffin wax and cut into 5-μm sections. Following deparaffinization and rehydration, the sections were processed for hematoxylin and eosin (H&E) staining and then examined. 

### 2.4. Gene Expression Analysis

The effect of MTX and FN on the mRNA expression levels of inducible nitric oxide synthase (*iNOS*), *BAX*, *BCL-2*, cyclooxygenase-2 (*COX-2*), *IL-1β*, *TNF-α*, *Nrf2*, *HO-1*, and caspase-3 was quantified using qRT-PCR, as previously reported [40,41,42]. Isolation of RNA from the frozen kidney samples was performed using TRIzol reagent (Invitrogen, Waltham, MA, USA). Following treatment with RNase-free DNase (Qiagen, Düsseldorf, Germany), RNA was quantified on a nanodrop, and samples with A260/A280 nm > 1.7 were reverse transcribed into cDNA. PCR amplification of the cDNA was carried out using SYBR Green master mix and the primers listed in Table 1. The amplification data were analyzed by the 2^−ΔΔCt^ method [43] and normalized to β-actin.

### 2.5. Western Blotting

Samples from the kidney were homogenized in ice-cold radioimmunoprecipitation assay (RIPA) buffer containing protease inhibitors, centrifuged, and protein content was determined in the supernatant using Bradford reagent. Forty micrograms of proteins were subjected to 10% SDS-PAGE followed by electrotransfer to a nitrocellulose membrane, which was blocked and probed with anti-Nrf2 and anti-β-actin. After overnight incubation at 4 °C and washing, the secondary antibodies were added, and the blots were developed. The obtained bands were scanned, and intensity was quantified using ImageJ (version 1.32 j, NIH, USA). The results were normalized to β-actin and presented as a percent of control. All antibodies were provided by Novus Biologicals (Centennial, CO, USA).

### 2.6. Assessment of the Impact of FN on MTX Cytotoxicity in HepG-2 Cells 

HepG2 cells were grown in PRMI-1640 supplemented with 10% fetal bovine serum (FBS), 1% glutamine and 1% penicillin/streptomycin (100 U/mL) at 37 °C and 5% CO_2_. Upon confluency, the cells were trypsanized and seeded in 96-well plates (10^4^ cells/well). The cells were treated with different doses of FN for 24 h, followed by MTX for 48 h. The cells were stained with 5 mg/mL MTT, incubated for 2 h at 37°C. The medium was replaced by 100 µl DMSO, and the absorbance was read at 570 nm after 10 min.

### 2.7. Statistical Analysis

The significance value of the obtained data was analyzed by one-way (ANOVA) followed by Tukey’s test using GraphPad Prism 7 (La Jolla, CA, USA). All results were presented as mean ± standard error of the mean (SEM). A *p*-value < 0.05 was considered significant.

## 3. Results:

### 3.1. FN Prevents Renal Dysfunction and Injury in MTX-Administered Rats

**Figure 1:**
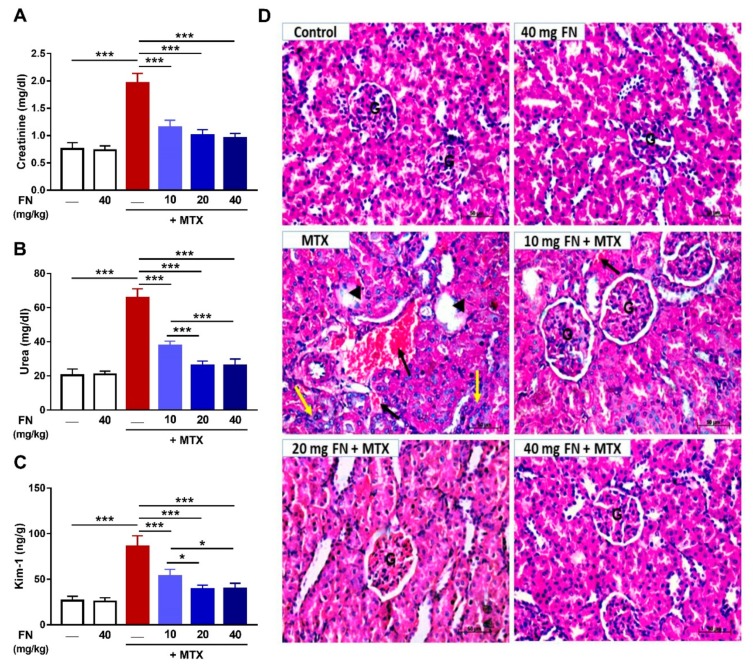
Formononetin (FN) prevents methotrexate (MTX)-induced renal dysfunction and injury. FN ameliorated serum creatinine (**A**) and urea (**B**) and renal kidney injury molecule-1 (Kim-1) (**C**) in MTX-administered rats. Data are mean ± SEM, (*n* = 6). *p < 0.05 and ***p < 0.001. (**D**) Photomicrographs showing the normal structure of the glomeruli (G) and renal tubules in control and FN-treated rats, and interstitial hemorrhage (black arrow), glomerular atrophy and necrosis (arrowhead), and infiltration of leukocytes (yellow arrow) in MTX-intoxicated rats. FN prevented kidney injury induced by MTX with interstitial hemorrhage (black arrow) observed at the 10 mg/kg dose. (hematoxylin and eosin (H&E); X400) [Scale bar = 50 µm].

MTX increased creatinine, urea, and Kim-1 significantly (*p* < 0.001) as depicted in Figure 1A–C. FN attenuated the MTX-induced kidney dysfunction, without altering the kidney function markers in normal rats (Figure 1A–C).

The renoprotective efficacy of FN was supported by the histological examination (Figure 1D). While the control and FN-supplemented animals showed normal renal tubules and corpuscles, MTX caused multiple alterations, including interstitial hemorrhage, glomerular atrophy, infiltration of leukocytes, and others. In contrast, rats which received 10, 20, and 40 mg/kg FN showed noticeable improvement in the kidney structure where all doses remarkably prevented MTX-induced tissue injury (Figure 1D).

### 3.2. FN Prevents Oxidative Stress and DNA Damage in MTX-Induced Rats

**Figure 2:**
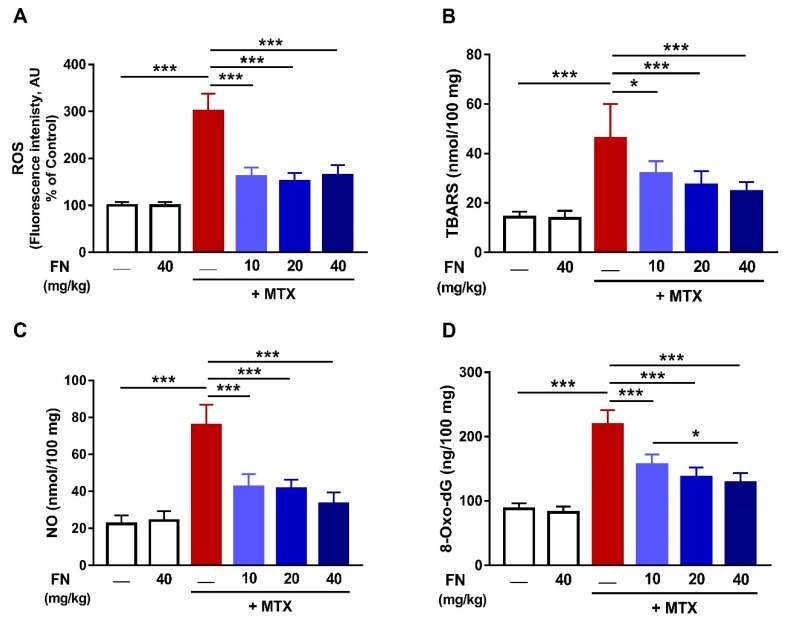
FN prevents oxidative stress and DNA damage in MTX-administered rats. FN reduced reactive oxygen species (ROS) (**A**), thiobarbituric acid reactive species (TBARS) (**B**), nitric oxide (NO) (**C**) and 8-Oxo-2′-deoxyguanosine (8-Oxo-dG) (**D**) in the kidney of rats which received MTX. Data are mean ± SEM, (*n* = 6). *p < 0.05 and ***p < 0.001.

The effect of FN on oxidative stress and DNA damage induced by MTX was assessed by determining ROS, LPO, NO, and 8-Oxo-dG. MTX triggered a significant increase (*p* < 0.001) in renal ROS (Figure 2A), LPO (Figure 2B), and NO (Figure 2C) levels. 8-Oxo-dG, a marker of DNA damage, was markedly increased in the kidney following MTX injection (Figure 2D). Rats received FN before MTX exhibited noticeable amelioration of renal ROS, LPO, NO, and 8-Oxo-dG levels.

**Figure 3:**
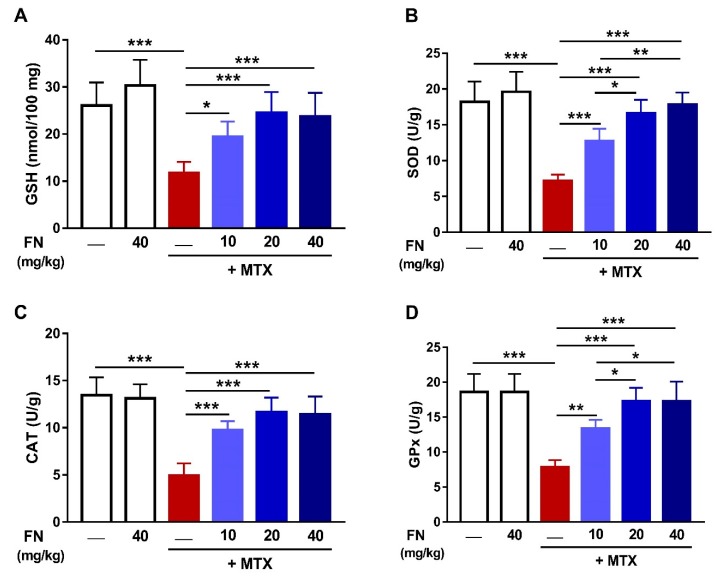
FN enhances antioxidants in the kidney of MTX-administered rats. FN increased renal glutathione (GSH) (**A**), superoxide dismutase (SOD) (**B**), catalase (CAT) (**C**), and glutathione peroxidase (GPx) (**D**) in MTX-induced rats. Data are mean ± SEM, (*n* = 6). * *p* < 0.05, **p < 0.01 and ***p < 0.001.

In addition to suppressing ROS generation and oxidative DNA damage elicited by MTX, FN boosted renal antioxidant defenses. MTX diminished renal GSH (Figure 3A), SOD (Figure 3B), CAT (Figure 3C), and GPx (Figure 3D) in rats. In contrast, animals received 10, 20, or 40 mg/kg FN before MTX exhibited markedly alleviated renal antioxidants. Of note, normal rats received FN showed no changes in both oxidative stress markers and antioxidants.

### 3.3. FN Upregulates Nrf2/HO-1 Signaling in Kidney of MTX-Administered Rats

**Figure 4:**
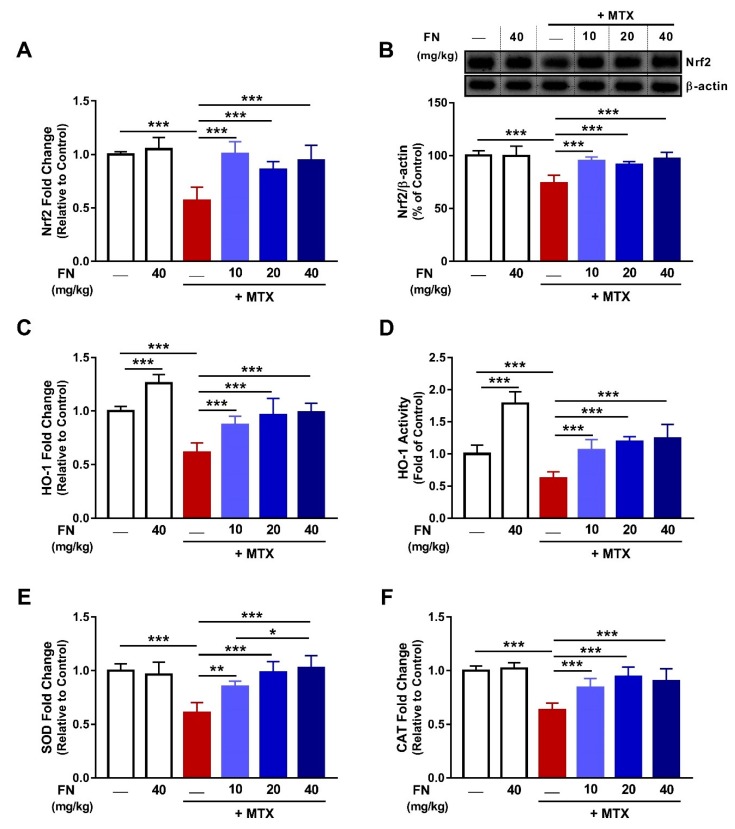
FN upregulates nuclear factor (erythroid-derived 2)-like 2/heme oxygenase 1 (Nrf2/HO-1) signaling in the kidney of MTX-administered rats. FN increased mRNA (**A**), and protein (**B**), levels of Nrf2, HO-1 mRNA (**C**), and activity (**D**), and mRNA of SOD (**E**) and CAT (**F**) in the kidney of rats received MTX. FN increased renal HO-1 mRNA (C) and activity (D) in normal rats. Data are mean ± SEM, (*n* = 6). *p < 0.05, **p < 0.01 and ***p < 0.001.

To explore the mechanism underlying the antioxidant efficacy of FN, Nrf2 expression, HO-1 both expression and activity, along with mRNA expression of SOD and CAT, were assessed. MTX downregulated renal Nrf2 in both the mRNA and protein in rats; an effect that was significantly attenuated by FN (Figure 4A,B). These data highlighted the MTX-induced suppression of Nrf2 signaling. This notion was confirmed by the diminished HO-1 mRNA (Figure 4C) and activity (Figure 4D), and mRNA abundance of SOD (Figure 4E) and CAT (Figure 4F) in the kidney of rats following MTX injection. FN (10, 20, and 40 mg/kg) prevented the deleterious effect of MTX on HO-1, SOD, and CAT. Although FN had no effect on Nrf2, SOD, and CAT in normal rats, it increased renal HO-1, both gene expression and activity.

### 3.4. FN Increases Renal ATP Levels in MTX-Induced Rats

**Figure 5:**
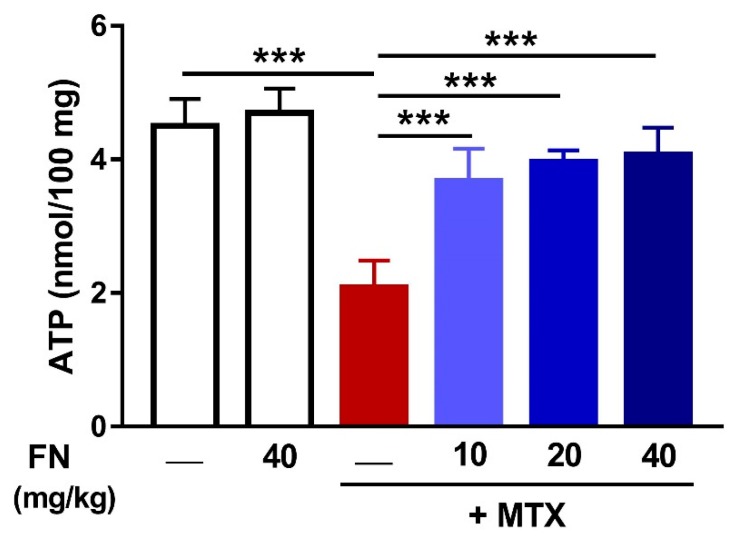
FN increases renal adenosine triphosphate (ATP) levels in MTX-induced rats. Data are mean ± SEM, (*n* = 6). ***p < 0.001.

Owing to the role of MTX in inducing mitochondrial dysfunction, we investigated the effect of MTX and FN on ATP content in the kidney. MTX diminished renal ATP (*p* < 0.001) and FN treatment prior to MTX restored mitochondrial function (Figure 5). FN alone had no effect on renal ATP of normal rats.

### 3.5. FN Suppresses Renal Inflammation in MTX-Induced Rats

**Figure 6:**
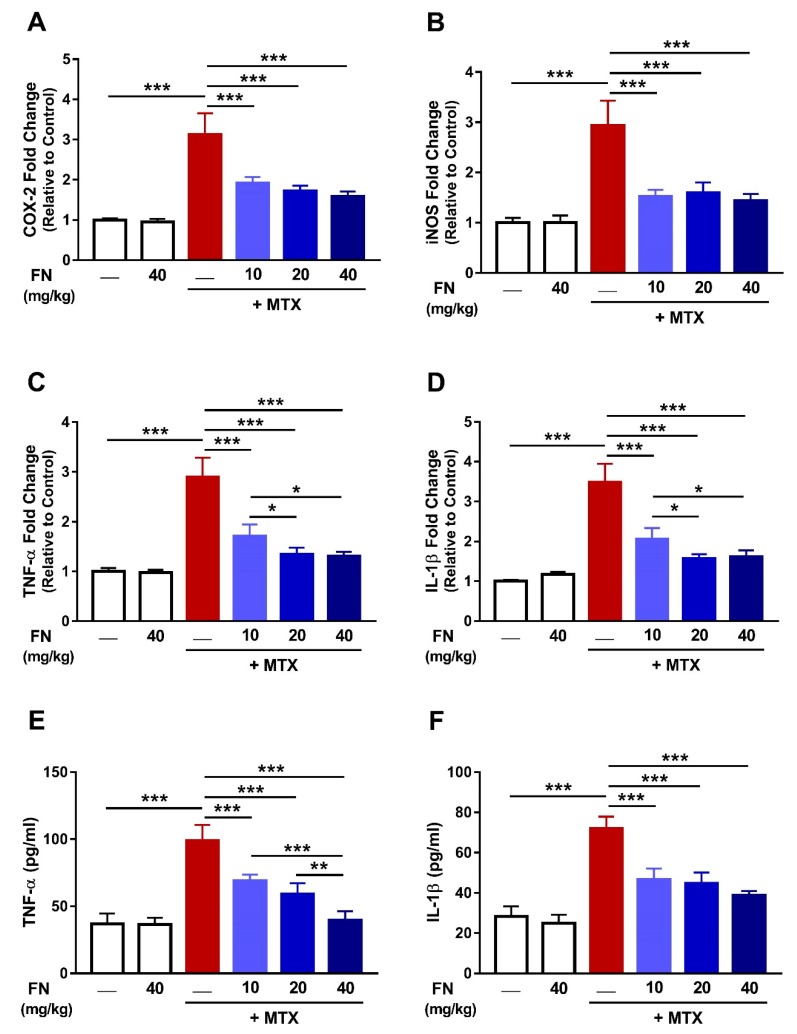
FN suppresses renal inflammation in MTX-induced rats. FN reduced mRNA abundance of renal COX-2 (**A**), iNOS (**B**), TNFα (**C**), and IL-1β (**D**), and serum TNFα (**E**) and IL-1β (**F**) in rats which received MTX. Data are mean ± SEM, (*n* = 6). *p < 0.05, **p < 0.01 and ***p < 0.001.

Analysis of the mRNA expression in the kidney of rats which received MTX revealed significant upregulation of COX-2, iNOS, TNF-α, and IL-1β (Figure 6). The inflammatory response following MTX administration was evidenced by the increased circulating levels of TNF-α and IL-1β. FN significantly attenuated the expression and circulating levels of the assayed inflammatory mediators and its effect on TNF-α and IL-1β mRNA and serum TNF-α was dose-dependent (Figure 6). All determined proinflammatory markers showed no changes in rats received 40 mg/kg FN.

### 3.6. FN Prevents MTX-Induced Apoptosis

**Figure 7:**
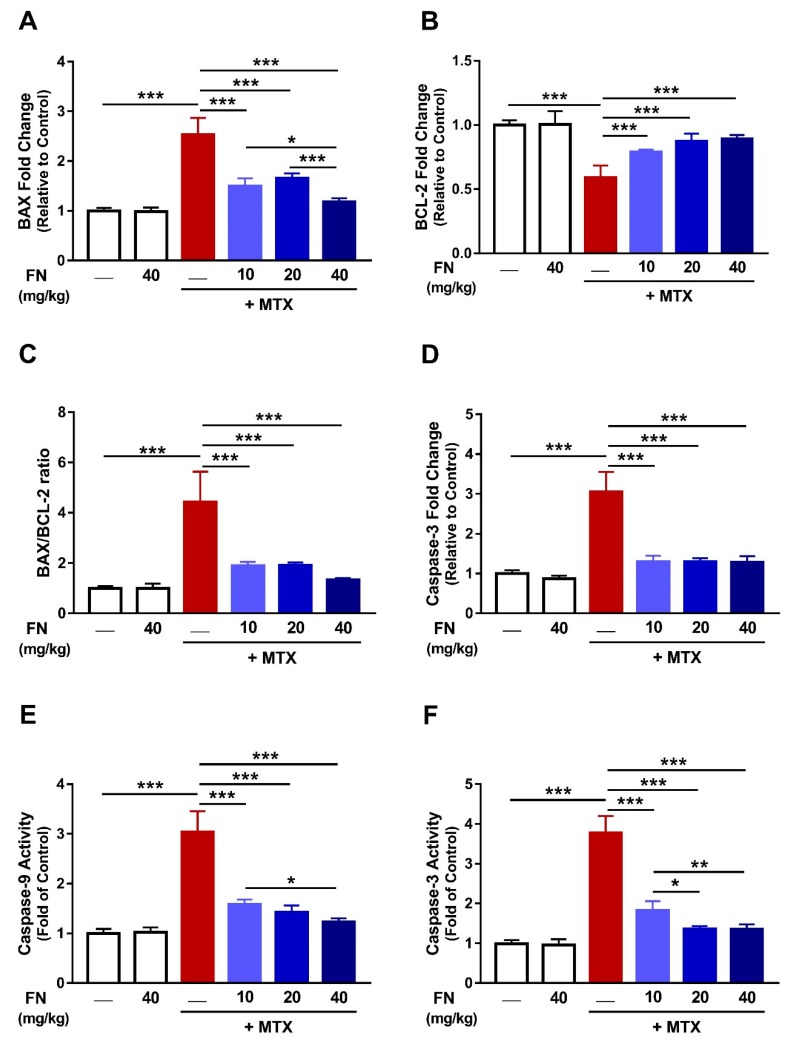
FN prevents MTX-induced apoptosis. FN decreased mRNA of Bax (**A**) and increased Bcl-2 expression (**B**) in the kidney of MTX-administered rats. Bax/Bcl-2 ratio (**C**), caspase-3 mRNA (**D**), caspase-9 activity (**E**), and caspase-3 activity (**F**) were decreased in rats which received FN before MTX. Data are mean ± SEM, (*n* = 6). *p < 0.05, **p < 0.01 and ***p < 0.001.

Oxidative damage, mitochondrial dysfunction, and inflammation can drive cell death. Given that MTX administration was associated with these processes, we determined its effect as well as the ameliorative potential of FN on apoptosis. MTX-mediated apoptosis was evidenced by the increased mRNA abundance of Bax (*p* < 0.001; Figure 7A) with a concomitant decline in Bcl-2 expression (Figure 7B) along with an increased Bax/Bcl-2 ratio (Figure 7C). Moreover, caspase-3 mRNA (Figure 7D) and activities of caspase-9 (Figure 7E) and caspase-3 (Figure 7F) were significantly increased following MTX injection. Remarkably, FN protected against MTX-induced apoptosis by diminishing Bax and caspases and enhancing Bcl-2 in rat kidney. FN had no effect on all apoptotic markers in normal animals (Figure 7).

### 3.7. FN Does Not Interfere with the Antitumor Activity of MTX

**Figure 8:**
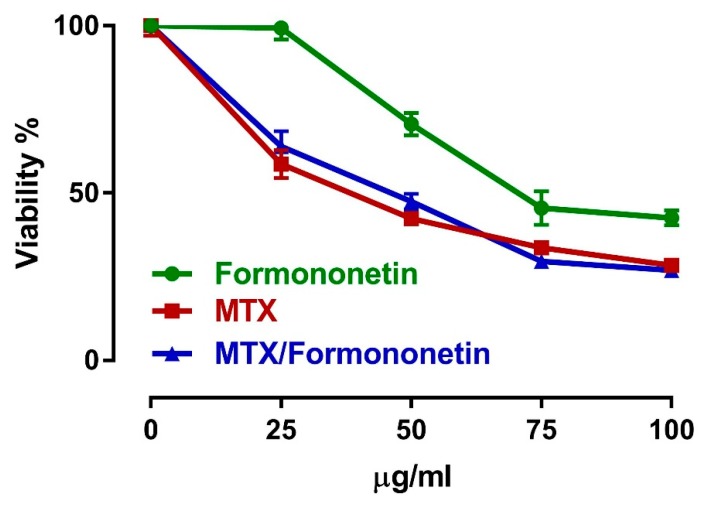
FN does not interfere with the antitumor activity of MTX. Treatment of HepG2 cells with either MTX or FN resulted in cytotoxicity. Per-treatment of HepG2 cells with FN did not interfere with the cytotoxic action of MTX. Data are mean ± SEM. The experiment was repeated three times (*N* = 3).

To assess the effects of FN on the antitumor properties of MTX, we investigated the cytotoxic effects of MTX alone and in combination with FN in HepG2 cells. Treatment of HepG2 cells with either MTX or FN resulted in cytotoxicity. Per-treatment of HepG2 cells with FN did not interfere with the cytotoxic action of MTX (Figure 8).

## 4. Discussion 

MTX is an effective anticancer and immunosuppressive agent; however, its serious side effects limit its clinical applications. The mechanism of MTX-induced nephrotoxicity involves oxidative injury and inflammation [6,7]. Despite the accumulating knowledge about MTX nephrotoxicity, efficient pharmacotherapies hampering this serious complication are unavailable. Hence, it is becoming more urgent to find novel therapeutic approaches to prevent and/or treat MTX nephrotoxicity. Herein, we investigated the protective effect of FN, a natural isoflavone with promising pharmacological activities, against MTX-induced AKI in rats. Our findings demonstrated that FN can effectively prevent renal oxidative injury, inflammation, and apoptosis in MTX-administered rats, possibly through augmenting Nrf2 signaling.

AKI in MTX-administered rats was evidenced by the elevated serum creatinine and urea, and renal Kim-1. Creatinine and urea are commonly measured as indices of glomerular function [44,45]. Kim-1 is a transmembrane protein markedly upregulated in renal injuries, particularly renal proximal tubules injury [46,47]. Therefore, elevated levels of these kidney injury biomarkers indicate renal dysfunction. AKI, induced by MTX, was further confirmed by the histological alterations. The examination of kidney sections revealed leukocyte infiltration, degenerative changes of glomeruli and tubular epithelial cells, interstitial hemorrhage, and others. These results were supported by previous studies where MTX administration was associated with altered renal function and histological structures [6,7,8]. Kidney injury is caused by the precipitation of MTX and its 7-hydroxy metabolite in the renal tubules, resulting in tubular obstruction and compromised renal clearance [3,5]. Remarkably, FN afforded protection and alleviated kidney function in MTX-intoxicated rats, indicating a potent renoprotective efficacy. FN effectively reduced creatinine, urea, and Kim-1 and prevented histological alterations induced by MTX. In accordance, FN has shown renoprotective effects in type 2 diabetes [29], and rhabdomyolysis- [48] and cisplatin-induced toxicity [24].

Oxidative stress is one of the main mechanisms through which MTX induces tissue damage [6,7,11,49]. MTX has been reported to increase ROS production by suppressing homocysteine remethylation, depletion of NADPH, stimulation of neutrophils, activation of NADPH oxidase, and mitochondrial dysfunction [9,10,11,13]. In turn, ROS can cause cell injury through oxidizing lipid and proteins, inactivating antioxidant enzymes, and provoking DNA damage, leading to a dysfunctional cellular protective response [50]. LPO can lead to loss of membrane integrity by altering its fluidity and permeability and inactivating membrane-bound receptors and enzymes. ROS also promote the oxidation of amino acid side chains and the formation of protein–protein cross-linkages, increasing the havoc throughout the cell [51]. Consistent with several previous studies [6,7], MTX increased renal ROS, LPO, and NO, and reduced GSH and antioxidant enzymes. NO, being derived from iNOS induction, can react with superoxide anion, forming peroxynitrite that, among other effects, modifies purine and pyrimidine bases, resulting in DNA breaks [52]. In accordance, our findings showed an increase in 8-Oxo-dG, a marker of DNA damage. Moreover, several lines of evidence suggested direct and indirect mitochondria-related toxicity as a common effector mechanism of nephrotoxicity. ROS can cause mitochondrial damage which, in turn, results in the formation of mitochondrial permeability transition pore, further resulting in altered oxidative phosphorylation and progressive depletion of ATP, which may eventually culminate in cell death [53]. Our results showed that MTX administration diminished ATP content in the kidney. Therefore, maintenance of the cellular redox balance can represent an effective strategy to prevent MTX-induced AKI.

FN has been reported to exert antioxidant actions in several preclinical models of a variety of pathological conditions associated with oxidative stress [24,26,29]. Hence, we assumed that the nephroprotective efficacy of FN could be attributed to its antioxidant activity. Our results showed attenuation of ROS generation and DNA damage, enhancement of cellular antioxidants, and restoration of mitochondrial function in MTX-administered rats. Thus, FN prevented MTX-provoked AKI via suppressing oxidative injury and restoring antioxidant defenses and mitochondrial function.

In addition to oxidative stress, MTX-induced ROS generation can elicit various stress signaling, such as NF-κB, which promotes the expression of TNF-α, IL-1β, COX-2, and iNOS [6,7,8,49]. Here, MTX upregulated COX-2 and iNOS, and this explained the increase in NO levels. MTX has also increased both gene expression and serum TNF-α and IL-1β, demonstrating an inflammatory response. During glomerular injury and tubulointerstitial diseases, NF-κB activation in podocytes, mesangial cells, and tubular cells has been reported [54,55]. Recent work from our lab demonstrated the activation of ROS/NF-κB/NLRP3 inflammasome axis in the kidney of rodents received MTX [8,49]. FN diminished the expression and circulating levels of proinflammatory mediators in MTX-intoxicated rats, demonstrating an anti-inflammatory activity. Consistently, FN attenuated neuroinflammatory reaction through downregulating TNF-α and IL-1β and upregulating IL-10 in a rat model of traumatic brain injury [56]. In rat insulinoma cell line, FN blocked IL-1β-induced NF-κB activation and consequent iNOS expression and NO production [28]. FN has also suppressed cognitive impairment in diabetic mice, possibly through the downregulation of TLR4/NF-κB signaling and NLRP3 inflammasome [56]. Given the recently reported role of ROS/NF-κB/NLRP3 inflammasome axis in MTX AKI [8,49], it could be assumed that suppression of this signaling pathway has a role in the anti-inflammatory effect of FN against MTX nephrotoxicity.

Accumulating evidence has pointed to the role of oxidative injury and inflammation in provoking apoptosis in MTX-induced AKI [6,7,8,49]. In this study, increased ROS and inflammation were associated with oxidative DNA damage and upregulated Bax and caspases. Bax is a proapoptotic protein that elicits cytochrome *c* release from the mitochondria and consequent activation of caspases. In addition, excessive mitochondrial ROS production during MTX metabolism can damage the mitochondrial membrane, resulting in the loss of membrane potential and consequently the release of cytochrome *c* which ultimately culminates in renal apoptosis by activating caspase-3 [13]. Thus, inhibition of MTX-mediated ROS generation and proinflammatory cytokines production can protect against apoptosis. Given its dual ability to attenuate oxidative injury and inflammation, FN prevented apoptosis in MTX-administered rats as shown by the diminished expression and/or activity of Bax and caspases and upregulation of the antiapoptotic Bcl-2. In the same context, previous studies have shown that FN suppressed apoptosis in rhabdomyolysis- [48] and cisplatin-induced nephrotoxicity [24] in rodents and cisplatin-induced LLC-PK1 cells [57].

To gain more insight into the potential underlying mechanism of the renoprotective effect of FN, we investigated the role of Nrf2/HO-1 signaling in mediating the effects of FN. Extensive evidence indicated that Nrf2 signaling plays comprehensive cytoprotective roles through activating the expression of several genes encoding detoxification, antioxidant, and anti-inflammatory proteins [6,14,17,58,59,60]. Therefore, pharmacological activation of the Nrf2 signaling may provide an additional protective strategy against chemotherapy-induced AKI. Herein, MTX diminished Nrf2 signaling as evidenced by the diminished Nrf2, HO-1, and other antioxidant enzymes. Consistently, we have recently demonstrated reduced renal Nrf2 and HO-1 expression in MTX-intoxicated rats [6,7]. Although exposure to moderate oxidative stress leads to Nrf2 activation, excessive and sustained ROS generation can diminish Nrf2 signaling in the kidney [6,7,8,49] and liver [4,18] of rats challenged with MTX. Thus, the diminished Nrf2/ HO-1 pathway is a direct consequence of the sustained ROS generation induced by MTX.

FN upregulated renal Nrf2 and consequent induction of HO-1, CAT, and SOD in rats challenged with MTX. Interestingly, FN increased HO-1 mRNA and activity in normal rats. Upregulated Nrf2 signaling by FN resulted in enhanced antioxidants and diminished ROS and oxidative damage. Furthermore, activation of Nrf2 had a key role in the anti-inflammatory and antiapoptotic effects of FN. Nrf2 and HO-1 can directly inhibit NF-κB signaling and proinflammatory cytokines, and activate the anti-inflammatory cytokines, thereby regulating the inflammatory cascade [61]. In this context, the lack of Nrf2 aggravated inflammation through activation of NF-κB and downstream proinflammatory mediators in murine cultured astrocytes [62], and the asperity of drug hepatotoxicity in mice [63]. The role of Nrf2 in mediating the anti-inflammatory and antiapoptotic efficacies of FN in MTX-administered rats was supported by previous reports which demonstrated increased expression of Nrf2 and suppressed inflammation in acetaminophen-induced hepatotoxicity [24], traumatic brain injury [64], and rhabdomyolysis-induced renal injury [48] in rodents. Another interesting finding in this study was the significant increase in HO-1 mRNA and activity in the kidney of normal rats.

To further investigate whether the above-mentioned beneficial effects of FN would interfere with the antitumor activity of MTX, we evaluated its effect on MTX-induced cytotoxicity in HepG2 cells. Per-treatment of HepG2 cells with FN did not interfere with the cytotoxic action of MTX. Indeed, several molecular mechanisms have been involved in the anticancer activity of MTX; however, oxidative/nitrative stress does not seem to be implicated. Efficient antioxidant strategies, which counteract the toxicity of chemotherapies, do not interfere with their antitumor action. For example, the cardioprotective agent dexrazoxane that reduces doxorubicin cardiotoxicity is a potent antioxidant [65]. Here, the renoprotective effect of FN did not interfere with the antitumor efficacy of MTX. In addition, FN by itself has also been demonstrated to exert various antitumor properties, including inhibition of AKT phosphorylation and induction of cervical cancer cell line HeLa apoptosis in a dose-dependent manner [66].

## 5. Conclusions

**Figure 9:**
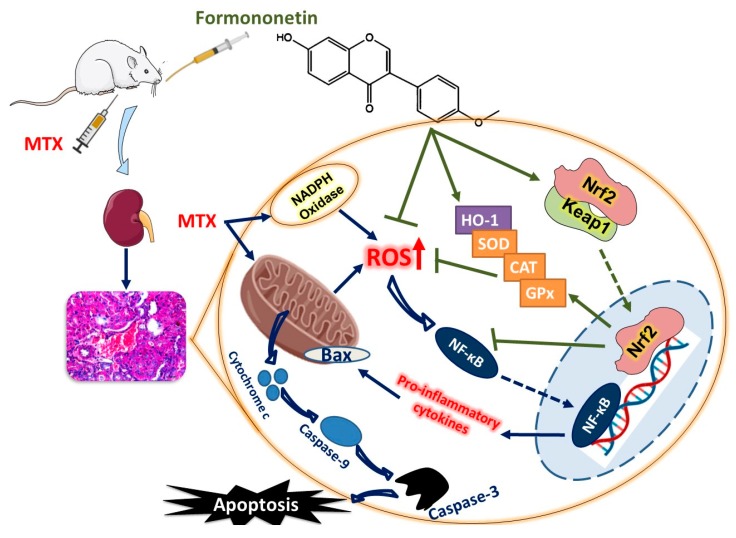
A schematic diagram illustrating the protective mechanism of FN against MTX nephrotoxicity. MTX provoked ROS generation, inflammation, and cell death in the kidney of rats. FN activated Nrf2/HO-1 signaling, boosted antioxidants, and prevented MTX-induced tissue injury.

Our findings indicate that the natural isoflavone FN prevented AKI induced by MTX. FN suppressed excess ROS, oxidative injury, and improved mitochondrial function. Consequently, FN attenuated inflammation and inhibited cell death in the kidney of rats and hence, possesses a therapeutic benefit against MTX toxicity. Activation of Nrf2/HO-1 signaling and enhancement of the antioxidant defenses represent the main mechanism underlying the nephrotprotective effect of FN (Figure 9). These results provide new information on the protective effects of FN against MTX-induced AKI. In addition, the reported antineoplastic properties of FN in various malignancies are particularly encouraging from the therapeutic point of view. However, the exact mechanism underlying the renoprotective action of FN undoubtedly deserves further exploration in upcoming studies.

## Figures and Tables

**Table 1:** Primers used for qRT-PCR.

Gene	Forward Primer (5′–3′)	Reverse Primer (5′–3′)
*NRF2*	TTGTAGATGACCATGAGTCGC	TGTCCTGCTGTATGCTGCTT
*HO-1*	GTAAATGCAGTGTTGGCCCC	ATGTGCCAGGCATCTCCTTC
*SOD*	ACACCTATGCACTCCACAGAC	ACATTCGACCTCTGGGGGTA
*CAT*	GCGGGAACCCAATAGGAGAT	CAGGTTAGGTGTGAGGGACA
*TNFa*	AAATGGGCTCCCTCTCATCAGTTC	TCTGCTTGGTGGTTTGCTACGAC
*IL-1b*	GACTTCACCATGGAACCCGT	GGAGACTGCCCATTCTCGAC
*COX2*	TGATCTACCCTCCCCACGTC	ACACACTCTGTTGTGCTCCC
*NOS2*	ATTCCCAGCCCAACAACACA	GCAGCTTGTCCAGGGATTCT
*BAX*	AGGACGCATCCACCAAGAAG	CAGTTGAAGTTGCCGTCTGC
*BCL2*	ACTCTTCAGGGATGGGGTGA	TGACATCTCCCTGTTGACGC
*Casp3*	GAGCTTGGAACGCGAAGAAA	TAACCGGGTGCGGTAGAGTA
*Actb*	AGGAGTACGATGAGTCCGGC	CGCAGCTCAGTAACAGTCCG
