# Correction: Aladaileh et al. Formononetin Upregulates Nrf2/HO-1 Signaling and Prevents Oxidative Stress, Inflammation and Kidney Injury in Methotrexate-Induced Rats. *Antioxidants* 2019, *8*, 430

**DOI:** 10.3390/antiox14091061

**Published:** 2025-08-29

**Authors:** Saleem H. Aladaileh, Omnia E. Hussein, Mohammad H. Abukhalil, Sultan A. M. Saghir, May Bin-Jumah, Manal A. Alfwuaires, Mousa O. Germoush, Amer A. Almaiman, Ayman M. Mahmoud

**Affiliations:** 1 Department of Medical Analysis, Princess Aisha Bint Al-Hussein Faculty of Nursing and Health Sciences, Al-Hussein Bin Talal University, Ma`an 71111, Jordan; sadaileh@ahu.edu.jo (S.H.A.); sultan.s.ayesh@ahu.edu.jo (S.A.M.S.); 2 Department of Biology, Faculty of Science, Al-Hussein Bin Talal University, Ma`an 71111, Jordan; 3 Physiology Division, Department of Zoology, Faculty of Science, Beni-Suef University, Beni-Suef 62514, Egypt; omniaaa411@yahoo.com; 4 Department of Biology, College of Science, Princess Nourah bint Abdulrahman University, Riyadh 84428, Saudi Arabia; mnbinjumah@pnu.edu.sa; 5 Department of Biology, Faculty of Science, King Faisal University, Al-Ahsa 31982, Saudi Arabia; malfwuaires@kfu.edu.sa; 6 Department of Biology, College of Science, Jouf University, Sakaka 2014, Saudi Arabia; germoush@ju.edu.sa; 7 Department of Applied Medical Sciences, Community College of Unaizah, Qassim University, Buraydah 51431, Saudi Arabia; ameralmeman@hotmail.com

In the original publication, there was a mistake in Figure 1 as published. Following the publication of this article [1], it has come to the authors’ attention that the image that represents the group 40 mg FN + MTX in Figure 1D was accidentally replaced by another image that belongs to a different group from an experiment that was conducted in parallel. The authors state that the scientific conclusions are unaffected. The updated Figure 1 is shown below. This correction was approved by the Academic Editor. The original publication has also been updated.

## Figures and Tables

**Figure 1:**
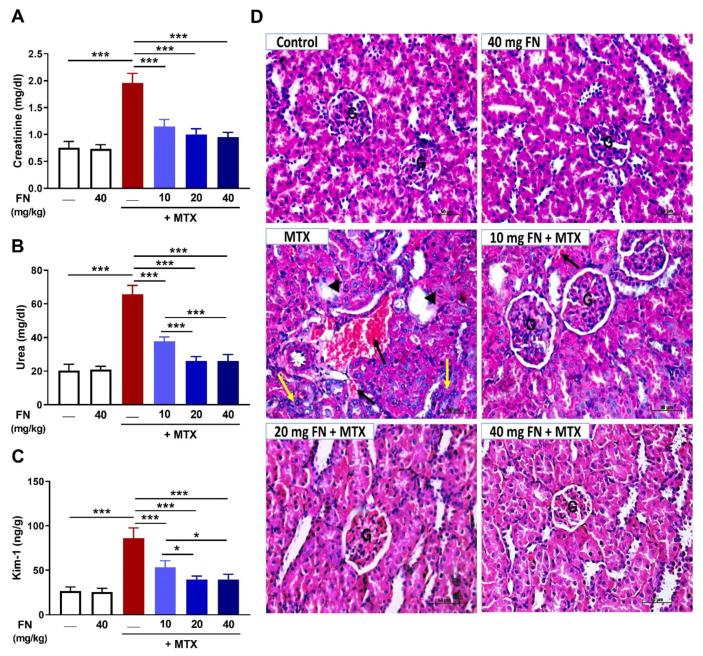
Formononetin (FN) prevents methotrexate (MTX)-induced renal dysfunction and injury. FN ameliorated serum creatinine (**A**) and urea (**B**) and renal kidney injury molecule-1 (Kim-1) (**C**) in MTX-administered rats. Data are mean ± SEM, (*n* = 6). * *p* < 0.05 and *** *p* < 0.001. (**D**) Photomicrographs showing the normal structure of the glomeruli (G) and renal tubules in control and FN-treated rats, and interstitial hemorrhage (black arrow), glomerular atrophy and necrosis (arrowhead), and infiltration of leukocytes (yellow arrow) in MTX-intoxicated rats. FN prevented kidney injury induced by MTX with interstitial hemorrhage (black arrow) observed at the 10 mg/kg dose (hematoxylin and eosin (H&E); X400) [Scale bar = 50 µm].
